# Clinical Validation and Treatment Plan Evaluation Based on Autodelineation of the Clinical Target Volume for Prostate Cancer Radiotherapy

**DOI:** 10.1177/15330338231164883

**Published:** 2023-03-29

**Authors:** Jing Shen, Yinjie Tao, Hui Guan, Hongnan Zhen, Lei He, Tingting Dong, Shaobin Wang, Yu Chen, Qi Chen, Zhikai Liu, Fuquan Zhang

**Affiliations:** 1 Department of Radiation Oncology, 34732 Peking Union Medical College Hospital, Beijing, China; 2 Radiotherapy Technique Development Department, MedMind Technology Co., Beijing, China

**Keywords:** prostate cancer radiotherapy, convolutional neural networks, clinical target volume, deep learning, DVH dosimetric index

## Abstract

**Purpose:**

Clinical target volumes (CTVs) and organs at risk (OARs) could be autocontoured to save workload. This study aimed to assess a convolutional neural network for automatic and accurate CTV and OARs in prostate cancer, while comparing possible treatment plans based on autocontouring CTV to clinical treatment plans.

**Methods:**

Computer tomography (CT) scans of 217 patients with locally advanced prostate cancer treated at our hospital were retrospectively collected and analyzed from January 2013 to January 2019. A deep learning-based method, CUNet, was used to delineate CTV and OARs. A training set of 195 CT scans and a test set of 28 CT scans were randomly chosen from the dataset. The mean Dice similarity coefficient (DSC), 95th percentile Hausdorff distance (95HD), and subjective evaluation were used to evaluate the performance of this strategy. Predetermined evaluation criteria were used to grade treatment plans, and percentage errors for clinical doses to the planned target volume (PTV) and OARs were calculated.

**Results:**

The mean DSC and 95HD values of the defined CTVs were (0.84 ± 0.05) and (5.04 ± 2.15) mm, respectively. The average delineation time was < 15 s for each patient's CT scan. The overall positive rates for clinicians A and B were 53.15% versus 46.85%, and 54.05% versus 45.95%, respectively (*P* > .05) when CTV outlines from CUNet were blindly chosen and compared with the ground truth (GT). Furthermore, 8 test patients were randomly chosen to design the predicted plan based on the autocontouring CTVs and OARs, demonstrating acceptable agreement with the clinical plan: average absolute dose differences in mean value of D2, D50, D98, Dmax, and Dmean for PTV were within 0.74%, and average absolute volume differences in mean value of V45 and V50 for OARs were within 3.4%.

**Conclusion:**

Our results revealed that the CTVs and OARs for prostate cancer defined by CUNet were close to the GT. CUNet could halve the time spent by radiation oncologists in contouring, demonstrating the potential of the novel autocontouring method.

**Note:** A preprint has been previously published.^1^

## Introduction

Prostate cancer is the commonest noncutaneous cancer in males, and has the second highest mortality among all cancers.^2^ Prostate cancer is expected to affect 1 in every 9 males at some point in their lives.^3^ Intensity-modulated radiation therapy (IMRT) is an important aspect of current cancer care for patients with prostate cancer; and with appropriate treatment, the 5-years survival rate is 98.2%.^4^ Radiation doses to the clinical target volume (CTV) and organs at risk (OARs) can often be significant and one of the essential steps for successful IMRT delivery owing to the complex dose distribution in IMRT. Laborious manual delineation is conventionally performed by radiotherapy oncologists for such delineation. However, this delineation is time-consuming.^5^ Therefore, automatic and accurate computational tools that could significantly lower the manual efforts put in by radiotherapy oncologists are warranted.

In recent years, the hoppoint convolutional neural networks (CNNs) have demonstrated their advantages in performing medical segmentation tasks,^6,8^ having been commonly used in the CTV or OARs delineation of brain cancer,^9^ head and neck (H&N) cancer,^10,13^ breast cancer, esophageal cancer,^14^ rectal cancer,^15^ and cervical cancer.^16^ U-Net, a well-known CNN architecture for medical image segmentation, was commonly used among all the CNN-based contour delineation models.^17^ These studies demonstrate that combining U-Net with an elegant feature extraction architecture would efficiently help obtain an effective CNN architecture for target region segmentation. On the other hand, attention has been paid to how to effectively improve network performance.^18^^–^^23^ The network could focus on important features and suppress unnecessary features via the attention mechanism. Herein, we integrate Convolutional Block Attention Module (CBAM) module that focus on meaningful features along the channel and spatial dimensions into U-Net to facilitate automatic CTV and OARs delineation.^24^

Intra-observer and interobserver variability is one of the major challenges in radiotherapy planning for prostate cancer.^25^ We further tried to design the clinical plan on autocontouring CTV and OARs, to compare its performance with clinical treatment plans, for exploring its clinical feasibility and accuracy. The autocontouring computed tomography (CT)-based dose prediction could decrease the time required for the iterative optimization and structural contouring, allowing physicians and dosimetrists to focus their expertise on more challenging cases. To the best of our knowledge, this is the first report to predict the dose distribution for IMRT using autocontouring deep learning CT images.

## Materials and Methods

### Patients Database

The Peking Union Medical College Hospital's Institutional Review Board ethnically approved the study in December 2021(approval number: K22C2234). The reporting of this study conforms to STROBE guidelines.^26^ CT data from 217 de-identified patients with locally advanced prostate cancer were retrospectively collected from January 2013 to January 2019. We statistically examined patient characteristics such as age and pathological type. The patients were given a total dose of 70 Gy in 28 fraction, using 6-Mv X ray of IMRT.

All the data were recorded using a Brilliance CT Big Bore with a size of 512 × 512 pixels (pixel spacing: ranging from 0.77 × 0.77 to 1.95 × 1.95 mm) and a 5 mm thickness (Philips Healthcare, Best, the Netherlands). Qualified radiation oncologists manually drew the CTV and OAR contours prior to irradiation in clinical practice as the segmentation ground truth (GT). Widely acknowledged consensus criteria with certain modifications were used to define the CTV.^27^ The prostate and seminal vesicle were combined to create the CTV. A professional radiation oncologist committee comprising 8 oncologists with > 10 years of experience in radiotherapy for pelvic tumors reviewed and modified (only when necessary) all of the delineated contours together, as is the clinical routine in our department, to ensure the quality of the delineation.

### Machine Learning Models Data and Preprocessing

The threshold of the supplied CT slice intensity was set between −1024 and 2048 HU. Zero-mean normalization was used to make the input picture data zero-centred and distributed in the same range for each patient, making training less sensitive to feature size and allowing for well-conditioned optimization. Patients from the dataset were randomly assigned to a training-validation set (195 patients) and a test set (28 patients).

**Figure 1.**
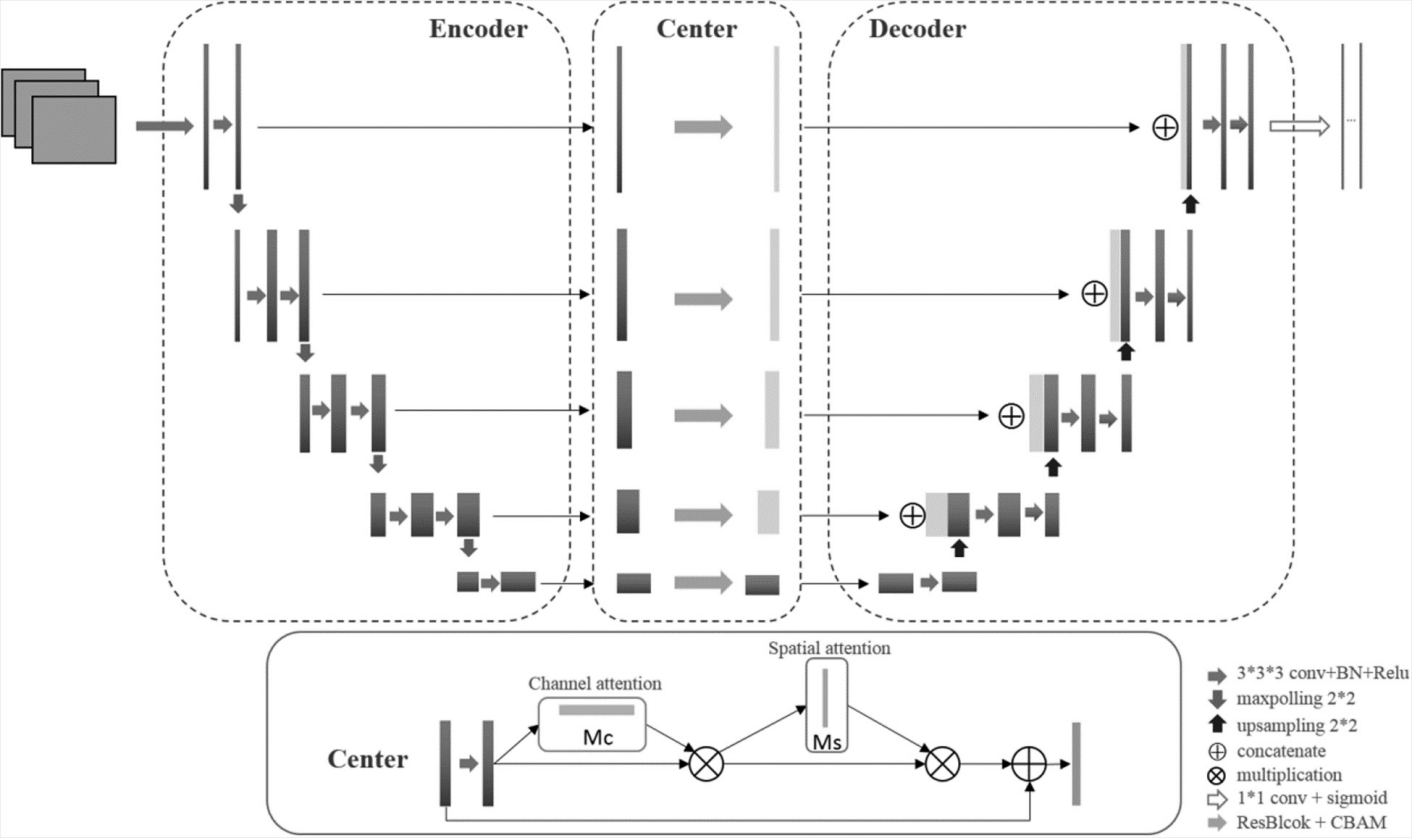
The architecture of the proposed CUNet.

The CUNet architecture is illustrated in Figure 1. Three-dimensional (3D) U-Net backbone architecture comprising encoding and decoding paths was used. The encoding and decoding paths were combined by a skip connection in the typical U-Net to concatenate the multilevel features, thus taking advantage of the low-level and high-level information. Thus, the network learns to use the features equally. An attention center block is proposed to weigh the features before concatenating them to the encoder part instead of combing the features directly in CUNet. Thus, the network will focus on specific features instead of all the features. The attention center block is a CBAM integrated with ResBlock in ResNet.^23^ CBAM infers attention maps along the channel and spatial dimensions sequentially. For adaptive feature refinement, each attention map is multiplied by the input feature map. A residual block skip connection then connects the final feature map with the initial input feature map. The channel attention map exploits the interchannel relationship of features, showing “what” is meaningful in terms of input image. The interspatial relationship of features generates the spatial attention map. The informative features will be emphasized while the other features will be suppressed.

The CTV, bladder, rectum, left femoral head, and right femoral head contours were trained using CUNet with 5 output channels.

### Model Training

Using a 5-fold cross-validation technique, 195 CT scans were used for training and validation. During training, patches with dimensions (64, 64, 64) were randomly generated and the model was trained from the scratch. Augmentation was not used. The output value of the model ranged from 0 to 1. The foreground of the segmented mask was set to pixels with an output value > 0.5. Following this, a contour extraction was given to the foreground. The network was trained on NVIDIA TITAN Xp (12 GB RAM). The Adam optimizer was used throughout the network, with an initial learning rate of 0.0001 and a decay rate of gamma 0.9 for each epoch. The total number of epochs was 50.

### Model Evaluation

#### Quantitative evaluation metrics

The Dice similarity coefficient (DSC) and 95th percentile Hausdorff distance (95HD) were used to assess the contouring accuracy. The volumetric overlap ratio between segmented masks is calculated using the DSC; a higher value indicates a higher overlap ratio. When 2 masks are totally identical, the value equals 1. The 95HD indicates the agreement between 2 outlines; a higher value suggests a greater divergence.

#### Oncologist evaluation

Red and green outlines were used to randomly mark the artificial intelligence (AI) and GT outlines respectively, in a clinically blinded assessment of drawing layers from 2237 slice layers of 28 patients, sorting after creating 111 layers. Two clinicians with > 10 years of experience in prostate cancer radiation blindly decided which contour was superior for clinical use. A positive outcome was recorded if the AI group performed better, and the number of AI layers that had better clinical applicability was reported.

### Plan Evaluation

Anticipated treatment plans based on autocontouring CTV and clinical plans were compared. The predicted dose was based on a reoptimized plan which was optimized based on the AI contours. The total dose-volume histogram (DVH) curves of planned target volume (PTV) and distinct OARs were first exhibited between the prediction and clinical truth plans in terms of DVH parameters. Second, the clinically relevant dosimetric indices (DI) were determined, including the mean dose (Dmean), D2, D50, and D98 for PTV (where Di represents the dose received by i percent of PTV volume) and Dmean, V45, and V50 for OARs (where Vi represents the volume fraction of OARs irradiated by i Gy). The following formula was used to calculate the homogeneity index and conformation index where Vptv and Vpres are the volumes of the PTV and the prescription dose region, respectively, and Vref is the volume of the irradiated PTV of the prescription dose.

$$HI=\frac{D2-D98}{D50}$$

$$CI=\frac{Vref*Vref}{Vptu*Vpres}$$

### Statistical Analysis

The CTV and OARs contours predicted from using CUNet were transferred to a treatment planning system for designing the radiotherapy plan. During radiotherapy planning, the total amount of time spent refining the CTV and OARs contours was documented in minutes per case. Per cent error relative to the prescribed dose was used to further evaluate the performance of the models, which was calculated as (predicted dose−clinical dose)/prescribed dose ×100%. The dosimetric parameters were indicated with median and interquartile range, and the parameters in clinical and predicted groups were compared by means of the Wilcoxon rank sum test. The significance between the average and maximum doses of the clinical and the predicted and mimicked plans of each models were investigated and analyzed. Statistical significance was set at *P* < .05 (2-tailed).

## Results

### Quantitative Analysis of the CTV and OAR Contour

CT data from 217 patients were enrolled, and 28 CT slices from the test dataset were analyzed. The mean DSC and 95HD values for the defined CTVs were (0.84 ± 0.05) and (5.04 ± 2.15) mm, respectively. The mean DSC and 95HD values for the OARs were reported in Table 1. The average delineation time was < 15 s for the CT scan of 1 patient.

**Table 1.** The Mean DSC and 95HD Values for OARs.

Anatomy	DSC ± STD	95HD ± STD
Bladder	0.913 ± 0.078	2.462 ± 3.984
Bone marrow	0.850 ± 0.051	1.973 ± 0.924
Femoral head left	0.899 ± 0.027	1.424 ± 0.329
Femoral head right	0.897 ± 0.023	1.547 ± 0.421
Rectum	0.783 ± 0.032	6.278 ± 2.275
Small intestine	0.822 ± 0.035	5.369 ± 2.294
Spinal cord	0.824 ± 0.056	3.345 ± 3.431

Abbreviations: 95HD, 95th percentile Hausdorff distance; DSC, Dice similarity coefficient; OAR, organs at risk; STD, sexually transmitted diseases.

### The Turing Imitation Test

A total of 10% of the slices with GT masks were chosen at random to mark both AI and GT contours simultaneously in the test dataset. The AI and GT CTV contours were demonstrated for each slice, with the color of these 2 contours randomly assigned as red or green. A total of 112 slices were chosen from the 28 CT images. The results are presented in Table 2.

**Table 2.** The Results of the Turing-Like Imitation Test.

	Clinician A		Clinician B	
	Slice	Ratio	Slice	Ratio
Positive	59	53.15%	60	54.05%
Negative	53	46.85%	52	45.95%
Total	112	*P* > .05	112	*P* > .05

**Figure 2.**
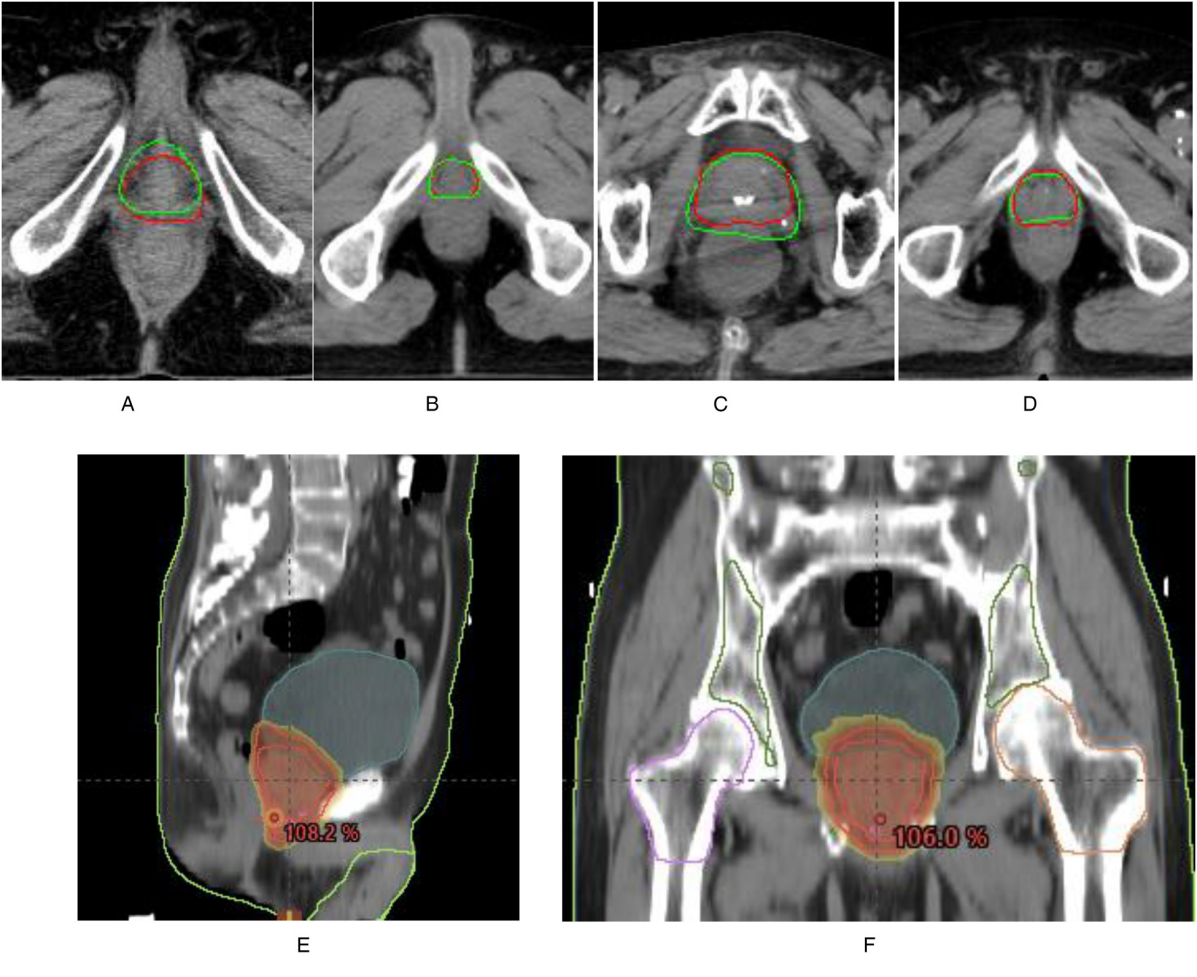
The distribution map of the positive results. (A) A sample slice wherein both the oncologists approved the GT contour. The GT contours are presented in green. AI contours are presented in red. (B) A sample slice wherein both oncologists approved the AI contours. The AI contours are presented in red. GT contours are presented in green. (C) A sample slice wherein only oncologist A approved the AI contour. AI contours are presented in green line. GT contours are presented in red. (D) A sample slice wherein only oncologist B approved the AI contour. AI contours are presented in green line. GT contours are presented in red. (E) and (F) Three-dimensional display of prostate contouring. Abbreviations: AI, artificial intelligence; GT, ground truth.

Subclass analysis was performed to evaluate the oncologists individually and the CT slice (Table 3). The sample CTV delineations are presented in Figure 2.

**Table 3.** The Subclass Analysis Results of the Turing-Like Imitation Test.

Clinician A	Clinician B		
	Negative	Positive	Total
Negative	28	25	53
Positive	24	35	59
Total	52	60	112

### Statistics of the DVH DI

Overall DVH comparisons of the PTVs and OARs between the clinical and predicted results for 8 randomly selected test participants, revealed that the clinical and anticipated DVHs of the PTV and OARs had an acceptable agreement for each patient.

The mean and standard deviation of the clinically relevant DI for PTV and OARs in the 8 test patients are shown in Table 4. The average absolute dose differences in mean value of D2, D50, D98, Dmax and Dmean for the PTVs are within 0.74%, and the average absolute volume differences in mean value of V45 and V50 for OARs are within 3.4%. The projected findings are comparable to the clinical truth without any statistical significance (*P* > .05).

**Table 4.** Median and Interquartile Range of DIs for PTV and OARs.

DI	Clinical	Prediction	*P* Value
PTV			
D98(Gy)	68.57 (1.31)	68.55 (1.63)	.65
D50(Gy)	71.84 (1.35)	71.73 (1.06)	.62
D2(Gy)	73.10 (1.59)	72.90 (1.24)	.51
Dmean(Gy)	71.67 (1.12)	71.53 (0.96)	.50
Dmax(Gy)	75.02 (2.04)	74.30 (1.04)	.18
HI	1.04 (0.03)	1.04 (0.03)	.96
CI	0.87 (0.006)	0.87 (0.009)	.57
Rectum			
V50(%)	14.47 (9.90)	13.48 (4.90)	.94
V45(%)	16.60 (11.39)	15.43 (3.97)	.95
D25(Gy)	28.03 (23.64)	26.51 (15.45)	.88
D50(Gy)	6.88 (6.38)	8.02 (5.32)	.63
Dmean(Gy)	18.93 (8.72)	18.56 (7.02)	.88
Bladder			
V50(%)	10.65 (11.95)	14.02 (14.48)	.64
V45(%)	12.10 (13.91)	16.15 (16.44)	.67
D25(Gy)	19.11 (27.89)	30.21 (33.00)	.67
D50(Gy)	5.17 (12.58)	9.93 (13.68)	.73
Dmean(Gy)	15.16 (13.89)	21.66 (16.86)	.70
Left femoral head			
D5(Gy)	16.42 (3.90)	16.16 (4.09)	.65
Dmean(Gy)	11.73 (5.23)	10.77 (4.50)	.98
Right femoral head			
D5(Gy)	16.29 (2.37)	16.99 (5.47)	.63
Dmean(Gy)	11.88 (3.38)	11.28 (4.48)	.72

Abbreviations: CI, conformation index; DI, dosimetric indices; Dmax, maximum dose; Dmean, mean dose; HI, homogeneity index; OAR, organs at risk; PTV, planned target volume.

## Discussion

Radiation oncology for prostate cancer is of significance because it can decrease its associated mortality. CTV and OAR contouring remains fundamental for treatment; however, it is time-consuming and prone to human errors, leading to potentially avoidable delays in treatment initiation. Currently, several CNNs have focused on autosegmenting prostate CTV contouring using a CNN-based method, and despite the satisfactory performance of the models, there is scope for improvement. There is a reasonably long way before these models can be used safely and effectively in clinical practice. Hence, we collected and standardized CT images from 237 patients in our department and proposed a new deep learning network, CUNet, to automatically segment the CTV and OARs in prostate cancer.

The mean DSC and 95HD values were used for comprehensively evaluating the performance of CUNet for prostate CTV contouring. The mean DSC and 95HD values for the delineated CTVs were 0.84 and 5.04 mm, respectively, demonstrating the highly quantitative CTV contours. The results were also acceptable for OARs delineation. Moreover, in the Turing imitation test, 2 clinicians with > 10 years of experience in radiotherapy for prostate tumors blindly selected which contour had better clinical applicability. The overall positive rate for clinicians A and B were 53.15% versus 46.85%, and 54.05% versus 45.95% (*P* > .05), demonstrating that our deep machine learning model performed equally well or even better than human delineation.

An inherent product of CTV autocontouring is a reduction in the time spent by the radiation oncologist on contouring. The average manual CTV contouring time for 1 patient with prostate cancer was 10 to 20 min,^28^ while the corresponding time was 15 s when CUNet was employed. Following this, an average time of 2 min was required for each case to refine the contours. Thus, it took approximately 8 min including 5 min for manual data transfer.

**Figure 3.**
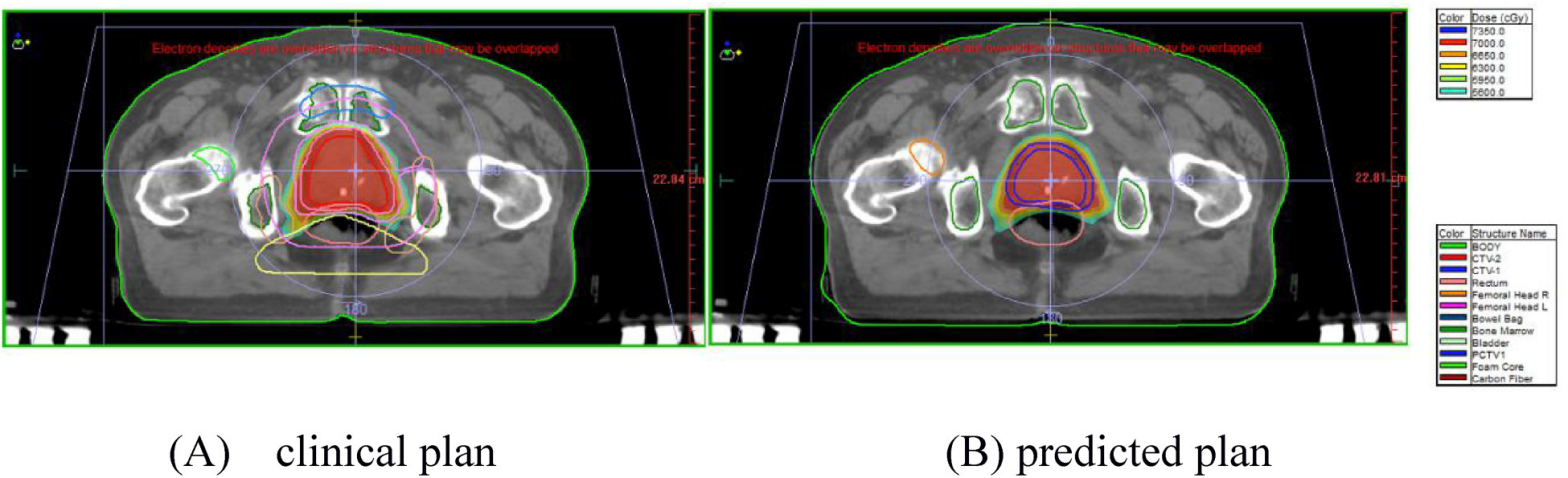
An example of the clinical and predicted plans. (A)The clinical and (B) predicted plans based on the autocontoured CTV in the same patient. Abbreviation: CTV, clinical target volumes.

The clinical and predicted (autocontouring) CTV treatment plans were further scored and compared. The overall DVH comparisons of the PTV and OARs for the 8 randomly selected test patients revealed that the average absolute dose differences of D2, D50, D98, Dmax, and Dmean for PTV were within 0.74%, and the average absolute volume differences of V45 and V50 for OARs were < 3.4%. The predicted results are comparable to the clinical truth, and no statistical significance was observed (*P* > .05) (comparison in Figure 3). All these results revealed that the dose distribution predictions using autocontouring CTV are accurate. Differences between the predicted doses for OARs of the models were lesser when compared with the clinical plans, which were not clinically relevant, thus demonstrating the potential of automated treatment planning for prostate cancer. The predicted dose distribution could serve as a quality control tool for treatment planning, wherein the planners can know whether or where the dose distributions can be improved, and the physicians can immediately visualize the 3D dose distributions to adjust the dose constraint requirements of the OARs. Meanwhile, the planners can take advantage of these OARs DVHs to define optimization, which improve the quality and consistency of treatment plans, and lower the planning time. However, this was still idealistic because it would take time for planners to make radiotherapy plans and new DVHs. In the future, this could be even faster if clinicians could get new DVHs automatically after contouring using the deep learning method.

Our study has several shortcomings. First of all, sample size was not well justified in the current study, and the number of DVHs included were small. Additionally, the majority of the participants were from Peking Union Medical College Hospital, where medical capacity is high, leading to possible selection bias. Further studies need to be carried out among medical researches in other regions and countries for comparisons of AI technology and manual contouring in different cultural contexts.

The current results for prostate CTV and OAR contouring demonstrate that CUNet can learn high-level semantic features well, making it possible to use this method for volume delineations in other cancers. It would form a sound foundation to further collect more data from multiple manufacturers and institutions for validating our study findings; however, this possibility warrants further research.

## Conclusions

Accurate and consistent delineation of the CTV and OARs is of significance in radiotherapy. This study presents a novel CNN (CUNet) for automatic and accurate CTV and OAR segmentation in prostate cancer. The performance of CUNet was quantitatively and subjectively evaluated for 28 patients. CUNet provided accurate delineation results, which had high clinical acceptability. The results demonstrate that CUNet can be used to improve efficiency and reduce the adverse effects associated with random errors in delineating the CTV and OARs in patients with prostate cancer.

## Abbreviations

3D: three-dimensional
95HD: 95th percentile Hausdorff distance
AI: artificial intelligence
CBAM: convolutional block attention module
CNN: convolutional neural network
CT: computed tomography
CTV: clinical target volumes
DI: dosimetric index
Dmean: mean dose
DSC: Dice similarity coefficient
DVH: dose-volume histogram
GT: ground truth
IMRT: intensity-modulated radiation therapy
OAR: organs at risk
PTV: planned target volume.

## Appendix 1. OARs’ Dose Constraints.

**Table:**

Normal Organs	Volume/Dose	Volume/Dose Constraints
Rectum	25%	≤3000 (cGy)
	35%	≤2500 (cGy)
	50%	≤2000 (cGy)
Bladder	25%	≤4000 (cGy)
	35%	≤3000 (cGy)
	50%	≤3000 (cGy)
Colon	25%	≤1000 (cGy)
	2cc	≤3500 (cGy)
Femoral head left	5%	≤2500 (cGy)
Femoral head right	5%	≤2500 (cGy)

Abbreviation: OAR, organs at risk.
